# Metagenomic Profiling of Soil Microbes to Mine Salt Stress Tolerance Genes

**DOI:** 10.3389/fmicb.2018.00159

**Published:** 2018-02-08

**Authors:** Vasim Ahmed, Manoj K. Verma, Shashank Gupta, Vibha Mandhan, Nar S. Chauhan

**Affiliations:** Department of Biochemistry, Maharshi Dayanand University, Rohtak, India

**Keywords:** metagenome, halotolerance, SSU rRNA, soil microbiome, soil ecology

## Abstract

Osmotolerance is one of the critical factors for successful survival and colonization of microbes in saline environments. Nonetheless, information about these osmotolerance mechanisms is still inadequate. Exploration of the saline soil microbiome for its community structure and novel genetic elements is likely to provide information on the mechanisms involved in osmoadaptation. The present study explores the saline soil microbiome for its native structure and novel genetic elements involved in osmoadaptation. 16S rRNA gene sequence analysis has indicated the dominance of halophilic/halotolerant phylotypes affiliated to *Proteobacteria, Actinobacteria, Gemmatimonadetes, Bacteroidetes, Firmicutes*, and *Acidobacteria*. A functional metagenomics approach led to the identification of osmotolerant clones SSR1, SSR4, SSR6, SSR2 harboring *BCAA_ABCtp, GSDH, STK_Pknb*, and *duf3445* genes. Furthermore, transposon mutagenesis, genetic, physiological and functional studies in close association has confirmed the role of these genes in osmotolerance. Enhancement in host osmotolerance possibly though the cytosolic accumulation of amino acids, reducing equivalents and osmolytes involving *BCAA-ABCtp, GSDH*, and *STKc_PknB*. Decoding of the genetic elements prevalent within these microbes can be exploited either as such for ameliorating soils or their genetically modified forms can assist crops to resist and survive in saline environment.

## Introduction

Soil is a rich and dynamic ecosystem, containing a vast number of microorganisms (van Veen et al., 1997). Geological activities like weathering of rocks, winds and poor agricultural practice are continuously increasing salt contents of the soils (Jiang et al., 2007; Canfora et al., 2014). Enhanced soil salinity modulates the microbial community structure and its physiological activity (Jiang et al., 2007; Canfora et al., 2014; Shrivastava and Kumar, 2015). The majority of microbes surviving in salt stress conditions demonstrate osmotolerance for varying duration, which may extend even to their entire lifespan (Roberts, 2005). The salt stress tolerance mechanisms are complex phenomena where pathways are coordinately linked (Culligan et al., 2012). These metabolic strengths to mitigate osmotic stress, seem to be genetically evolved through horizontal gene transfer (Koonin and Wolf, 2012; Yan et al., 2015; Gupta et al., 2017). Description of these osmotolerance mechanisms is crucial for comprehensive understanding of the biology of saline soil microbes, and exploiting them for their applications in improving soil quality and crop yields (Xiao and Roberts, 2010; Zhengbin et al., 2011; Culligan et al., 2012, 2013, 2014; Fernandes, 2014). A variety of culture dependent studies have been carried out to decode the gene(s) involved in osmotolerance within halophilic or halotolerant microbes (Zuleta et al., 2003; Klähn et al., 2009; Naughton et al., 2009; Meena et al., 2017). These studies have deciphered the role of proteins, Na^+^/H^+^ pumps, compatible solutes in salt stress tolerance (Sakamoto and Murata, 2002; Roberts, 2005). However, culture independent approach provides a vast opportunity for searching salt tolerant gene (s) (Singh J. et al., 2009; Mirete et al., 2015; Kumar J. et al., 2016; Chauhan et al., 2017; Gupta et al., 2017). Only a few studies have used metagenomic approach to decode the microbial salt stress tolerance mechanisms from various environments like pond water (Kapardar et al., 2010a,b), brines and moderate-salinity rhizosphere (Mirete et al., 2015), human gut microbiome (Culligan et al., 2012, 2013, 2014). Incidentally, the number of genes/pathways identified for salt stress tolerance are far below than the number of microbes which have been identified to reside within these environments (Humbert et al., 2009; Mirete et al., 2015). Hereby, the current study was proposed to identify the genetic machinery used by microbes as survival strategies in salt stress condition using functional metagenomic approach. The current study led to the identification of a number of osmotolerant genes that could be used to develop strategies to ensure survival of microbes under saline conditions.

## Materials and methods

### Saline soil sample collection and metagenomic DNA isolation

Saline soil samples were collected in sterile containers after carefully removing the surface layer (up to 10 cm) from Village Malab, District Nuh situated at 28.0107°N, 77.0564°E. Metagenomic DNA was extracted from 5 g of the soil sample (Supplementary Methods).

### Bacterial strains and growth conditions

Bacterial strains and plasmids used in the study are listed in Table 1. The oligonucleotides used in the study (GeNoRime, Shrimpex Biotech services Pvt. Ltd. India) are listed in Supplementary Table S1. *Escherichia coli* (DH10B) and *E. coli* (MKH13) strains were cultured in Luria-Bertani (LB) medium. Further, *E. coli* (DH10B) and *E. coli* (MKH13) strains containing *pUC19* vector were cultured in LB medium supplemented with ampicillin (100 μg ml^−1^). All overnight cultures were grown in LB broth at 37°C with constant shaking at 200 rpm.

**Table 1:** Bacterial strains and plasmids used in present study.

**Strains, plasmids, and transposons**	**Genotype or characteristics**	**Source or reference**
*E. coli* (DH10B)	F- *endA1 recA1 galE15 galK16 nupG rpsL* Δ*lacX74* Φ80*lacZ*ΔM15 *araD*139 Δ(*ara, leu*)7697 *mcrA* Δ(*mrr-hsdRMS*-*mcrBC*) λ-	Lucigen corporation, Parmenter St. Middleton, USA
*E. coli* (MKH13)	MC4100Δ(*putPA*)101D(*proP*)2D(*proU*)	(Haardt et al., 1995)
*pSSR1*	*pUC19* harboring a metagenomic DNA fragment of 2939 bp	Present study
SSR1	*E. coli* (DH10B) containing *pSSR1*	Present study
*pSSR4*	*pUC19* harboring a metagenomic DNA fragment of 2945 bp	Present study
SSR4	*E. coli* (DH10B) containing *pSSR4*	Present study
*pSSR6*	*pUC19* harboring a metagenomic DNA fragment of 1456 bp	Present study
SSR6	*E. coli* (DH10B) containing *pSSR6*	Present study
*pSSR21*	*pUC19* harboring a metagenomic DNA fragment of 2352 bp	Present study
SSR21	*E. coli* (DH10B) containing *pSSR21*	Present study
*pSSR1C1*	*pUC19* recombinant plasmid harboring putative *BCAA_ABCTP* of *pSSR1* cloned at *EcoR1* and *HindIII of pUC19 MCS (Multiple cloning site)*	Present study
SSR1C1	*E. coli* (MKH13) harboring *pSSR1C1*	Present study
*pSSR4C1*	*pUC19* recombinant plasmid harboring putative *GSDH* of *pSSR4* cloned at *EcoR1* and *HindIII of pUC19 MCS*	Present study
SSR4C1	*E. coli* (MKH13) harboring *pSSR4C1*	Present study
*pSSR21C1*	*pUC19* recombinant plasmid harboring putative *duf3445* gene of *pSSR21* cloned at *EcoR1* and *HindIII of pUC19 MCS*	Present study
SSR21C1	*E. coli* (MKH13) harboring *pSSR21C1*	Present study
*pUC19*	Plasmid cloning vector Amp^r^	Thermo Scientific
Transposon EZ Tn5^TM^ <Kan-2>	Tn5^TM^ Transposon Kan^r^	Epicenter Biotechnologies Madison, Wisconsins, USA

### Phylogenetic reconstruction of saline soil metagenome

Saline soil metagenomic DNA was used to amplify the SSU rRNA gene (Supplementary Methods). The amplified product was used for next generation sequencing (NGS) with the aid of Roche 454 GS FLX+ platform (Morowitz et al., 2011; Gupta et al., 2017). Finally, Quantitative Insights Into Microbial Ecology (QIIME) 1.9.0 pipeline was implemented for SSU rRNA sequence data analysis (Caporaso et al., 2011). SSU rRNA gene sequence data was curated for quality, length and ambiguous bases as a quality filtering step. Each sample was pre-processed to remove sequences with length less than 200 nucleotides and more than 1,000 nucleotides and sequences with minimum average quality <25. Reads with ambiguities and barcode mismatch were discarded. Reads were assigned to operational taxonomic units (OTUs) using a closed reference OTU picking protocol using QIIME. The uclust was applied to search sequences against a subset of the Greengenes database, version 13_8 filtered at 97% sequence identity. The OTUs were classified taxonomically by using the Greengenes reference database at various taxonomic ranks (phylum, class, order, family, genus, and species).

### Metagenomic library screening and characterization of salt resistant clones

Plasmid borne saline soil metagenomic library was prepared in *E. coli* DH10B using *pUC*19 vector (Supplementary Methods) (Chauhan et al., 2009, 2017) and manually screened for salt stress tolerant clones (Kapardar et al., 2010a,b). Salt stress resistant clones were screened by plating the soil metagenomic library (~165,000 clones with an average insert of 1.89 Kb) on LB agar medium supplemented with ampicillin (100 μg ml^−1^) and NaCl [5.8% (w/v)]. The 5.8% of NaCl (w/w) is a lethal concentration for *E. coli* DH10B cells and will allow the growth of only osmotolerant clones. RFLP analysis of salt stress tolerant clones was performed after digesting their recombinant plasmid DNA with *Eco*RI & *Hin*dIII at 37°C for 12 h. The minimum inhibitory concentration assay and growth inhibition studies were performed to analyze the salt stress tolerance property (Kapardar et al., 2010a,b). Growth inhibition assays of salt sensitive *E. coli* MKH13 clones were performed with 3% NaCl (w/v) & 3.7% KCl (w/v), while 5.8% NaCl (w/v) & 5.5 % of KCl (w/v) were used for *E. coli* DH10B clones. Graphs (created using Origin61) are presented as the average of triplicate experiments, with error bars being representative of the standard error of the mean.

### Genetic and physiological characterization of salt tolerance genes

The plasmid insert from salt resistant recombinant clones were sequenced using Sanger sequencing chemistry with primer walking approach at Eurofins Genomics India Pvt. Ltd (Bangalore, India). Sequence assembly was performed with Seq-Man sequence assembly software Lasergene package, version 5.07 (DNA Star, USA). Putative open reading frame (ORF) was predicted using an ORF finder tool at NCBI (http://www.ncbi.nlm.nih.gov/gorf/gorf.html) and checked for the database homology with Basic Local Alignment and Search Tool (BLAST) (http://www.ncbi.nlm.nih.gov/blast). Encoded protein sequences were analyzed for the presence of conserved domains (CDD) (Marchler-Bauer et al., 2014), topology prediction (HMMTOP) (Tusnády and Simon, 2001), phylogenetic analysis (MEGA7) (Kumar S. et al., 2016), and various physiological parameters (Tsirigos et al., 2015). Transposon mutagenesis of *pSSR1, pSSR4, pSSR6*, and *pSSR21* was carried out with EZ-Tn5™ <Kan-2> Insertion kit (Epicenter Biotechnologies) following manufacturer's instructions. Transposon mutants of *pSSR1, pSSR4, pSSR6*, and *pSSR21* were screened for the salt stress resistant and sensitive phenotypes to identify the active osmotolerant genomic regions within the cloned DNA fragment in *pSSR1, pSSR4, pSSR6*, and *pSSR21*. Salt tolerant active loci encoding putative *BCAA-ABCtp, GSDH, STK_Pknb*, and *DUF3445* genes of *pSSR1, pSSR4, pSSR6*, and *pSSR21* were subcloned in *pUC19* vector (*E. coli* MKH13 host) using standard molecular cloning techniques. The growth studies of subclones were performed to analyze their salt stress maintenance property in the presence of salt stressors NaCl [3.0% (w/v)] and KCl [3.7% (w/v)]. All assays were performed in triplicates for calculation of standard deviation. A parametric *t*-test was used to calculate the *p*-value.

### Elemental quantification of Na^+^ in salt tolerant clones

Elemental Quantification of intracellular Na^+^ in *E. coli* MKH13 carrying the empty vector (*pUC*19) and salt tolerant recombinant subclones (SSR1C1, SSR4C1, SSR6C1, SSR21C1) was measured with inductively coupled plasma spectroscopy-atomic emission spectroscopy (ICP-AES) analysis (Mirete et al., 2015) at SAIF, IIT Bombay, India. Results were expressed as mg of Na^+^ g^−1^ dry weight of cells. A parametric *t*-test was used to calculate the *p*-value.

### Data availability

Sequence reads generate in present study has been deposited in the NCBI SRA under accession number SRS2727172.

## Results

### Phylogenetic reconstruction of saline soil metagenome

**Figure 1:**
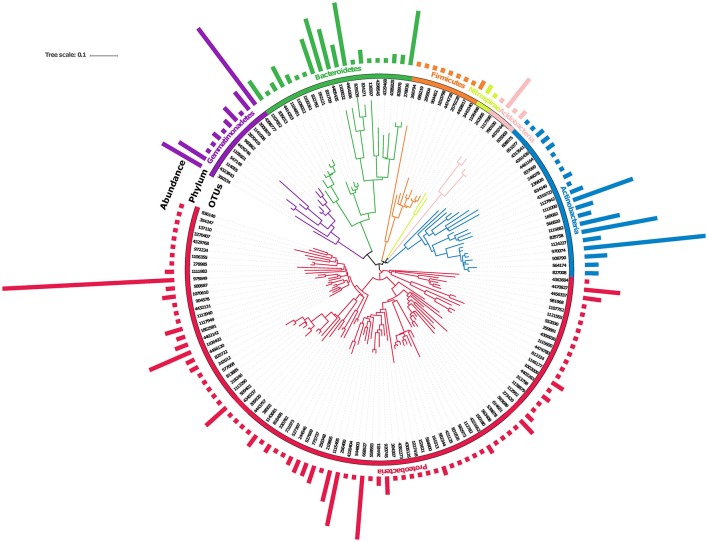
Cladogram of operational taxonomic units (OTUs) identified by SSU rRNA gene sequence analysis.

Physico-chemical properties of saline soil showed that the pH of soil was 9.0 ± 0.025, while its electrical conductivity (EC) was 6.5 ± 0.023. Elemental analysis of soil showed the excessive presence of salts, sodium (105 ppm), potassium (155 ppm), and lithium (188 ppm), confirming its moderate saline nature. A good quality (A260/280 >1.8), high molecular weight (>23 Kb) metagenomic DNA was extracted from the saline soil sample. Saline soil metagenomic DNA was used to analyze its SSU rRNA gene sequences to decode its native microbiome structure. Clustering of SSU rRNA gene identified a total of 487 OTUs distributed across seven microbial phyla (Figure 1). Out of 487 OTUs, we observed 153 unique OTUs (Supplementary Table S2). The inferred phylogeny of the soil microbiome based upon 153 unique OTUs (Figure 1) was comparable to the taxonomic classifications against with greengenes database, with most of the diversity of the microbiome being attributed to phyla *Proteobacteria*. The phylogeny was visualized by iTOL (Letunic and Bork, 2016). The dominant microbial phyla were *Proteobacteria, Actinobacteria, Gemmatimonadetes*, B*acteroidetes, Firmicutes*, and *Acidobacteria* (Figure 1). Among these phyla, the majority of sequences were affiliated to Proteobacteria (43.7%) having a representation of *Alphaproteobacteria* (38.9%), *Betaproteobacteria* (7%), *Deltaproteobacteria* (10.7%), and *Gammaproteobacteria* (43.2%); followed by Actinobacteria (21.8%) showing presence of *Acidimicrobiia* (72.47%) and *Nitriliruptoria* (18.8%); Bacteroidetes (18.1%) having a proportionate representation of *Rhodothermi* (51.9%), *Flavobacteriia* (25.96%), *Cytophagia* (19.3%), and Gemmatimonadetes (11.3%) with a percentage representation of *Gemm-2* (60.17%), *Gemm-4* (12.3%), *Gemm-1* (3.5%). Simultaneously, a minor fraction of sequences was affiliated to *Acidobacteria* (2.5%), *Firmicutes* (2.1%), and *Nitrospirae* (0.6%) microbial groups within saline soil microbiome. The taxonomic classification of saline soil microbiome confirms that a majority of microbial taxa belongs to phylum *Proteobacteria* (Figure 1) (Supplementary Table S2).

### Screening of salt stress resistant clones from saline soil microbiome

**Figure 2:**
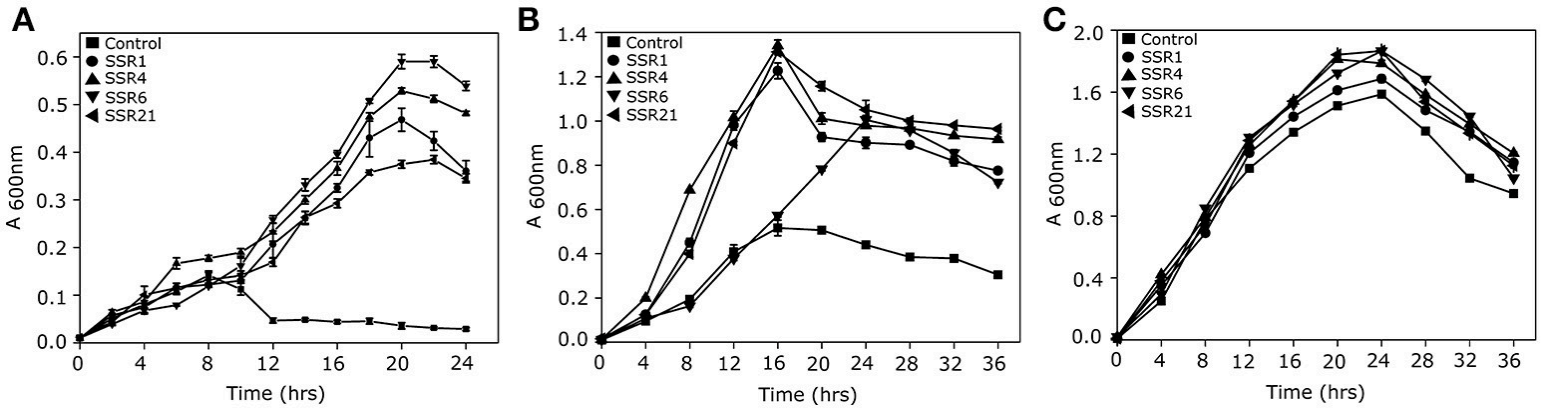
Characterization of salt stress tolerant clones for osmotolerance property. Growth of *E. coli* (DH10B) metagenomic clones SSR1 (•), SSR4 (▴), SSR6 (▾),SSR21(◂), and *E. coli* (DH10B) host strain carrying empty plasmid vector (■) in **(A)** LB broth supplemented with 4.0% NaCl (w/v), **(B)** LB broth supplemented with 5.5% KCl and **(C)** LB broth.

**Figure 3:**
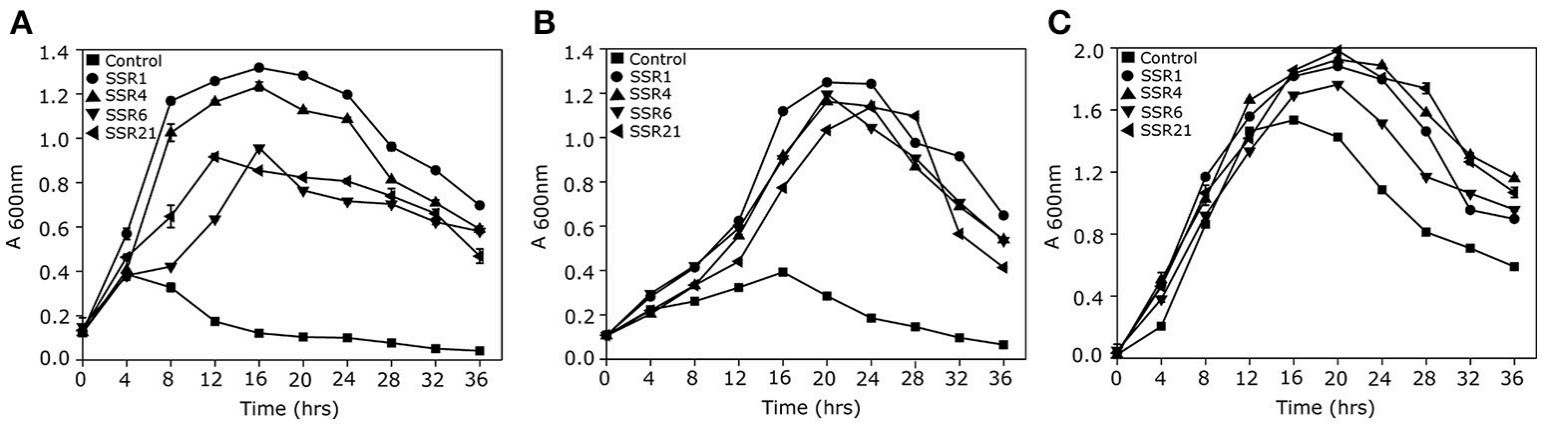
Complementation of osmotolerance property of salt stress tolerant clones. Growth of *E. coli* (MKH13) metagenomic clones harboring *pSSR1* (•), *pSSR4*(▴), *pSSR6*(▾), *pSSR21*(◂), and *E. coli* (MKH13) host strain carrying empty plasmid vector (■) in **(A)** LB broth supplemented with 3.0% NaCl(w/v), **(B)** LB broth supplemented with 3.7% KCl,**(C)** LB broth.

A saline soil metagenomic library was constructed with a total representation of 312 MB of cloned soil microbiome DNA. Primary screening of a saline soil metagenomic library at 5.8% NaCl (w/v) led to the identification of 24 salt stress tolerant clones. However, RFLP analysis indicated the presence of only four unique recombinant plasmids, labeled as *pSSR1, pSSR4, pSSR6*, and *pSSR21*. Minimum inhibitory concentration analysis showed almost two fold higher salt stress tolerance of SSR1, SSR4, SSR6, and SSR21 clones in comparison to the control *E. coli* (DH10B) (Supplementary Figure S1). The SSR1, SSR4, SSR6, and SSR21 also showed a statistically significant (*P* = 0.0009, *P* = 0.0003, *P* = 0.0014, *P* = 0.004) growth advantage in the presence of NaCl [4.0% (w/v)] (Figure 2A) and KCl [5.5% (w/v)] (*P* = 0.0045, *P* = 0.0008, *P* = 0.0486, *P* = 0.0022) as compared to *E. coli* (DH10B) strain carrying empty plasmid vector (*pUC*19) (Figure 2B), whereas no significant growth difference was observed between SSR1, SSR4, SSR6, SSR21, and native host *E. coli* (DH10B) carrying *pUC*19 in the presence of LB broth only (Figure 2C). Simultaneously *pSSR1, pSSR4, pSSR6*, and *pSSR21* successfully complemented salt stress tolerance property within salt sensitive *E. coli* (MKH13) strain and showed a statistically significant growth advantage in the presence of NaCl [3.0% (w/v)] (*P* = 0.0007, *P* = 0.0002, *P* = 0.0003, *P* = 0.0001) (Figure 3A) and KCl [3.7% (w/v)] (*P* = 0.0003, *P* = 0.0008, *P* = 0.0003, *P* = 0.0023) (Figure 3B), as compared to *E. coli* (MKH13) strain carrying empty plasmid vector (*pUC*19). At the same time, no significant growth difference was observed between *E. coli* (MKH13) strain harboring *pSSR1, pSSR4, pSSR6, pSSR21* as compared to *E. coli* (MKH13) strain carrying empty plasmid vector in the presence of LB broth only (Figure 3C).

### Genetic and physiological characterization of salt stress tolerant clones

#### Salt tolerant clone SSR1

**Figure 4:**
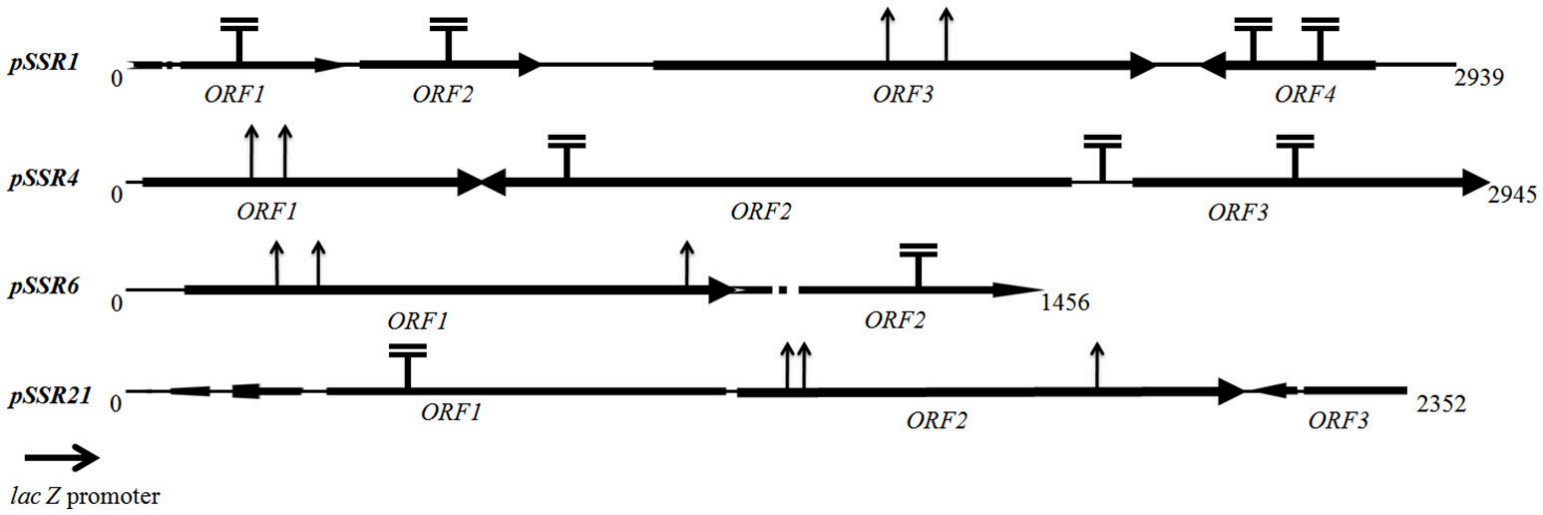
Transposon insertion map of *pSSR1, pSSR4, pSSR6*, and *pSSR21*. $\bar{\text{T}}$ indicates a transposon insertion site identified within transposon positive mutants (no effect on plasmid derived osmotolerance property) while ↑ indicate transposon insertion site identified within transposon negative mutants (loss of plasmid derived osmotolerance property).

**Figure 5:**
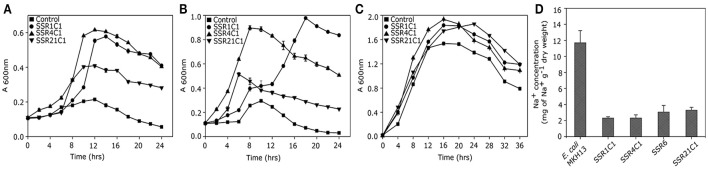
Physiological Characterization of *BCAA_ABCTP, GSDH, STK_Pknb*, and *duf3445* for osmotolerance. Growth curve analysis of osmotolerant phenotype SSR1C1 (•), SSR4C1 (▴), SSR21C1 (▾), and *E. coli* (MKH13) host strain carrying empty plasmid vector (■) in **(A)** LB broth supplemented with 3% NaCl (w/v), **(B)** LB broth supplemented with 3.7 % KCl (w/v), and **(C)** LB only. Intracellular Na^+^ estimation in *E. coli* (MKH13) strain harboring *pSSR1C1, pSSR4C1, pSSR6*, and *pSSR21C1* and *E. coli* (MKH13) host strain carrying empty plasmid vector **(D)**.

Sequence assembly of *pSSR1* resulted into a contig of 2,938 bp with a 66.87% G+C content. Cloned insert shared 76% homology with a halophilic proteobacterial lineage *Haliangium ochraceum*, indicating its plausible affiliation within proteobacterial clade. The gene prediction analysis indicated the presence of three complete and one truncated *ORF*, encoding proteins of 93, 114, 383, and 149aa respectively. Translated nucleotide sequences of these ORFs were subjected to BLASTP (maximum *e*-value cutoff of 1e-34) analysis to identify the homologous sequence in the database (Table 2). Transposon mutagenesis analysis confirmed functionally active locus for osmotolerance property encompassing *ORF3* (positioned between 927 and 2,078 bp) (Figure 4). *ORF3* encoded transmembrane protein shared homology with a transmembrane ABC transporter ATP-binding protein of *Betaproteobacteria bacterium SG8_39* (74%) and branched-chain amino acid ABC transporter substrate-binding protein of *Oceanibacterium hippocampi* (71%). Pfam database search identified the presence of a periplasmic ligand-binding domain of the ABC (ATPase Binding Cassette) type active transport systems, known to be involved in the transport of three branched chain aliphatic amino acids (leucine, isoleucine and valine) (Davidson et al., 2008). STRING analysis also predicted ORF3 as part of an interactive periplasmic binding protein dependent transport system. Further, NsitePred web server identified strong nucleotide binding sites (ATP Binding site at Gly14 and ADP binding site at Gly12) within *ORF3* encoded protein. This nucleotide binding site could be NBD, a common feature for ATP binding proteins, as predicted by its functional assignment. In consideration of physiological role and all structural features of *ORF3* encoded protein, it is a type of ABC transporter ATP-binding protein involved in salt stress maintenance possibly through energy dependent interaction with ABC membrane transporters involved in the exchange of the solutes across membrane, thus labeled as putative branched chain amino acid (BCAA) ABC transporter gene (*BCAA_ABCTP*). The putative *BCAA_ABCTP* gene was subcloned (*pSSR1C1*) to confirm its osmotolerance property. Time dependent growth curve analysis of SSR1C1 harboring *BCAA_ABCTP* showed a significant growth advantage in the presence of NaCl [3.0% (w/v)] (*P* = 0.0006) (Figure 5A) and KCl [3.7 % (w/v)] (*P* = 0.0005) (Figure 5B) as compared to salt sensitive *E. coli* mutant MKH13 carrying only the empty vector (*pUC*19), while no significant difference was observed on LB only (Figure 5C). The intracellular elemental analysis in the presence of ionic stressor NaCl [3.0% (w/v)] showed that SSR1C1 has effectively reduced intracellular sodium ion concentration (*P* = 0.0134) in comparison to *E. coli* mutant MKH13 (Figure 5D). A reduced intracellular sodium concentration within SSR1C1 could be seen as a result of its transporter property, as predicated through genetic characterization. The growth pattern of SSR1C1 was found similar to the native SSR1 that confirms that salt tolerance of salt tolerant clone SSR1 was due to SSR1C1 cloned insert encoding a branched chain amino acid (BCAA) ABC transporter protein.

**Table 2:** Open reading frames identified in recombinant plasmids of osmotolerant clones.

	**ORF details (ORF number, location, frame, product size)**	**Database homolog (accession no.)**	**Organism**	**Identity (%)**	**Coverage (%)**	***e*-value**	**Conserved domain**
*pSSR1* (2938bp 66.87%)	ORF1, 13–293^*^+1, 93 aa	ABC transporter ATP-binding protein (LivF) (WP_056712341)	*Bosea* sp. *Leaf344*	81	94	5e-51	ATP-binding cassette transporter nucleotide-binding domain (PRK13786)
	ORF2, 305–649, +2, 114aa	Hypothetical Membrane protein (WP_037447688)	*Skermanella stibiiresistens*	58	95	2e-43	–
	ORF3, 927–2078, +2, 383aa	ABC transporter (KPK07993)	*Betaproteobacteria bacterium* SG8_39	74	93	0.0	Type I periplasmic ligand-binding domain of ABC (cd06342)
	ORF4, 2617–2168, −1, 149aa	Transcriptional regulator (OJX81021)	*Magnetospirillum* sp. 64–120	71	91	1e-76	ROS/MUCR transcriptional regulator protein (pfam05443)
*pSSR4* (2945bp 63.63%)	ORF1, 21–644, +3, 207aa	Hypothetical protein AMJ56_20605 (KPK02757)	*Anaerolineae* bacterium SG8_19	64	99	1e-91	Glucose/Sorbosone dehydrogenase (GSDH) (pfam07995)
		Glucose/sorbosone dehydrogenase-like protein (WP_011340324)	*Pelobacter carbinolicus*	54	98	6e-69	
	ORF2, 2161–638, −2, 507aa	Hypothetical protein (CUR52060)	Uncultured Crenarchaeote	36	47	7e-47	mcbC-like_oxidoreductase (cd02142)
	ORF3, 2312 -2945, +2, 210aa	DUF262 domain-containing protein (WP_081445540)	*Actinomyces odontolyticus*	26	30	0.010	DUF4131 (pfam13567)
*pSSR6* (1456bp 65.659%)	ORF1, (108–1145), +3, 345aa	Serine/threonine-protein kinase (WP_082990042)	*Woeseia oceani*	81	100	0.0	Catalytic domain of bacterial Serine/Threonine kinases, PknB and similar proteins (cd14014)
	ORF2, (1121–1456^*^), +2, 111aa	Serine/threonine phosphatase (WP_068616118)	*Woeseia oceani*	90	85	2e-59	PP2Cc Superfamily (cd00143)
*pSSR21* (2352bp 69.98%)	ORF1, 913–2^*^, −3, 309aa	No Similarity	–	–	–	–	–
	ORF2, 912–1925, +3, 337aa	hypothetical protein A3F84_26310 (OGG46007)	*Candidatus Handelsmanbacteria* RIFCSPLOWO2_12_FULL_64_10	51	97	7e-113	DUF3445 (pfam11927)
	ORF3, 2097–2351^*^, −2, 84aa	Pilus assembly protein PilF (OFW28714)	*Acidobacteria* bacterium RIFCSPLOWO2_12_FULL_60_22	53	98	4e-20	TPR_10 (pfam13374)

* *Indicates truncated ORF*.

#### Salt tolerant clone SSR4

The *pSSR4* harbors a G+C rich (G+C% = 63.63) insert of 2,945 bp. The cloned sequence did not share any homology at the nucleotide level in existing database sequences. A total of three *ORF*s were predicted within the cloned insert, encoding proteins of 207, 507, and 210 amino acids respectively (Table 2). Transposon mutagenesis analysis identified the functionally active locus within the sequence region (235 bp), encompassing *ORF1* (Figure 4). *ORF1* encodes a cytosolic protein homologous to hypothetical protein of *Anaerolineae* bacterium SG8_19 and Glucose/sorbosone dehydrogenase-like protein of *Pelobacter carbinolicus*. A Pfam analysis of *ORF1* encoded protein indicates it as a glucose/sorbosone dehydrogenase protein having conserved domains for the protein family GSDH. It indicates that the *ORF1* possibly encodes a glucose/sorbosone dehydrogenase involved in salt stress tolerance, possibly through the cytosolic accumulation of reducing equivalents (NADPH and GSH). Predicted G*SDH* gene was amplified from *pSSR4* and subcloned to validate its osmotolerance property. Time dependent growth curve assay of SSR4C1 showed a significant growth advantage NaCl [3.0% (w/v)] (*P* = 0.0004) (Figure 5A) and KCl [3.7 % (w/v)] (*P* = 0.0006) (Figure 5B) as compared to salt sensitive *E. coli* mutant MKH13 carrying the empty vector (*pUC*19), while no significant difference has been observed on LB only (Figure 5C). The growth pattern of SSR4C1 was similar to SSR4. It also confirmed that the salt tolerance property of salt tolerant clone *pSSR4* was possibly due to *GSDH* gene encoding glucose/sorbosone dehydrogenase protein.

#### Salt tolerant clone SSR6

Sequence assembly of *pSSR6* generated a G+C rich (65.66%) contig of 1,456 bp. The blastn analysis identified its low similarity to *Betaproteobacteria* GR16-43 genome sequences, indicating its affiliation from proteobacterial clade. The cloned sequence encodes only one complete *ORF* (*ORF1*), encoding a cytosolic protein of 345 amino acids with a G+C content of 65.79% (Table 2). Transposon mutagenesis analysis also confirmed the functionally active locus within *ORF2* (Figure 4). Homologs of translated *ORF2*, corresponds to putative serine/threonine protein kinase of *Woeseia oceani* and *Mycobacterium smegmatis* str. MC2 155. A pfam database search of *ORF*2 encoded protein indicates it as a putative serine/threonine protein kinase having conserved domains for the protein family STKc_PknB, i.e., the catalytic domain of bacterial Serine/Threonine kinases, PknB family. Ser/Thr protein kinase homologs were found to be involved in osmosensory signaling in microbes (Hatzios et al., 2013). These identified proteins were important for the survival and in stress responses (Donat et al., 2009). The intracellular elemental analysis in presence of the ionic stressor NaCl [3.0% (w/v)] showed that SSR6 effectively reduced intracellular sodium ion concentration (*P* = 0.0200) in comparison to *E. coli* MKH13 (Figure 5D). A reduced intracellular sodium concentration within SSR6C1 could be due to enhanced ion transporter activity under the influence of signals generated by putative STKc_PknB of *pSSR6* under the osmotic stress.

#### Salt tolerant clone SSR21

The *pSSR21* was found to have a G+C rich (69.98%) insert of 2352 bp. The blastn analysis identified its homology with an *Actinomycetes* strain *Allokutzneria albata*. Gene prediction has indicated the presence of three *ORFs* in pSSR21, encoding proteins of 309, 337, and 84 amino acids respectively. Among three identified *ORF*s, only *ORF*2 was complete, while other two ORFs (ORF1 and 3) were truncated. Transposon mutagenesis analysis has identified the functionally active locus within *ORF2* sequence region (1,220 and 1,330 bp). The database homologs of *ORF*2 corresponds to hypothetical protein A3F84_26310 of *Candidatus Handelsmanbacteria* bacterium (Table 2). The pfam analysis identified the presence of conserved domains in the protein family DUF3445, i.e., protein of unknown function (DUF3445). The G+C content of *ORF2* was found to be 69.96% and the predicted functionally active region, *ORF2* was subcloned. Time dependent growth curve assay of SSR21C1 showed a significant growth advantage NaCl [3.0% (w/v)] (*P* = 0.0005) (Figure 5A) and KCl [3.7% (w/v)] (*P* = 0.0045) (Figure 5B) as compared to salt sensitive *E. coli* mutant MKH13 carrying only the empty vector (*pUC*19), while no significant difference has been observed on LB only (Figure 5C). Elemental Quantification of intracellular Na^+^ in *E. coli* MKH13 carrying the empty vector and salt tolerant recombinant subclones SSR21C1 clearly showed that the cloned gene insert (*duf3445*) within SSR21C1 has significantly reduced the concentration of intracellular Na^+^ ion (*P* = 0.0174) (Figure 5D).

## Discussion

Metagenomics has the potential to advance our knowledge by studying the genetic components of uncultured microbes (Singh A. H. et al., 2009; Mirete et al., 2015; Chauhan et al., 2017; Yadav et al., 2017). Looking at the perspectives of metagenomics, it was used to explore soil microbiome for its composition and genetic/physiological mechanisms allowing successful adaptation of microbes in saline environments. Simultaneously, these genes could be utilized as potential candidate to develop ever demanding drought resistant transgenic crops (Zhengbin et al., 2011) or osmotolerant microbes for food processing (Fernandes, 2014) and waste water treatment applications (Xiao and Roberts, 2010). Metagenomic analysis based on SSU rRNA gene has identified dominance of *Proteobacteria, Actinobacteria, Bacteroidetes*, and *Gemmatimonadetes* in saline soil microbiome. These results are in parallel with the outcome of previous studies defining microbial community composition of saline soil environment (Zhang et al., 2003; Ma and Gong, 2013; Canfora et al., 2014; Kadam and Chuan, 2016). Canfora et al. has reported a correlation in abundance of a microbial group with respect to soil salinity. They had indicated a relative abundance of *Proteobacteria* with *Bacteroidetes* were positively and *Acidobacteria* was negatively correlated with salinity (Canfora et al., 2014). Similarly *Actinobacteria* was also been reported as another dominant microbial phylum in saline ecosystems (Kadam and Chuan, 2016)*. Gemmatimonadetes* is another well-known hypersaline microbial phylum associated with biogeochemical transformations (Zhang et al., 2003). The existence of halophilic subcomponents within the identified microbial phylum, possibly making them capable to proliferate in a saline environment. These studies explain the abundance of *Proteobacteria, Actinobacteria, Gemmatimonadetes*, and *Bacteroidetes* in the studied ecosystem. Evolution of novel genetic features is a key to their successful survival in a changing environment (Gupta et al., 2017; Kumar Mondal et al., 2017). These saline microorganisms could have evolved salt stress tolerant genetic machinery to adapt and survive under salt induced osmotic stresses (Meena et al., 2017). A number of genetic elements were decoded for osmotolerance property from cultured and uncultured microbial representatives (Kapardar et al., 2010a,b; Culligan et al., 2012, 2013, 2014; Kim and Yu, 2012; Mirete et al., 2015). However, there is a disparity in a number of reported salt tolerance genes with a number of microorganisms from an ecosystem (Humbert et al., 2009; Mirete et al., 2015). This divergence could be allied with a number of factors like unculturability (Kim and Yu, 2012), lack of good quality genomic/metagenomic DNA (Kumar J. et al., 2016) and issues with foreign gene expression (Prakash and Taylor, 2012). An additional effort was made in the current study to decode osmotolerance genes prevalent in these saline soil microorganisms using functional metagenomics. It leads to the identification of four unique osmotolerant clones harboring DNA insert showing affiliation within halophile genomes. Genetic and physiological analysis has identified genes encoding putative proteins like membrane bound branched chain amino acid (BCAA) ABC transporter protein (BCAA_ABCTP), Glucose/sorbosone dehydrogenase, cytosolic STKc_PknB and DUF protein are responsible for osmotolerance property within salt stress tolerant clones. Among these, the role of the branched chain amino acid (BCAA) ABC transporter protein and glucose/sorbosone dehydrogenase in osmotolerance are well documented (Takami et al., 2002; Brosnan and Brosnan, 2006), while the scanty information is available about role for STKc_PknB and DUF3445 protein in stress maintenance (Hatzios et al., 2013).

ABC branched chain amino acid (BCAA) transporters are widely distributed in various marine microbes like *Oceanibacillus iheyensis* (Takami et al., 2002), *Salinispora, Bacillus*, and *Roseobacter* strains (Penn and Jensen, 2012). BCAA_ABCTP proteins are involved in the transport of branched chain aliphatic amino acids such as leucine, isoleucine and valine at high salt concentration. In the presence of 2-oxoglutarate and pyridoxal-5-phosphate, these branched chain amino acids are further converted to L-glutamate by branched chain amino acid transferase (Hutson, 2001). Accumulated glutamate acts as an osmoprotectant upon hyper-osmotic shock and activates sets of genes that allow the host to achieve long-term adaptation to high osmolarity (Gralla and Vargas, 2006). They also account for a significant proportion of the genes observed in the marine metagenome. Branched chain amino acid transporters are probably an important marine adaptation because accumulated glutamate may function as a counter ion for K^+^, which balances the electrical state of the cytoplasm (Penn and Jensen, 2012). Previous studies have reported a regulatory relationship between K^+^ and glutamate accumulation in response to osmotic stress in enteric bacteria and haloalkaliphilic archaea *Natronococcus occultus* (Kokoeva et al., 2002). Similarly, *T. consotensis*, a halotolerant bacterium accumulates glutamate to maintain electrical equilibrium within the cell in response to high salt concentrations (Rubiano-Labrador et al., 2015). This background information explains the possible physiological role of the BCAA_ABCTP gene of *pSSR1* to increase host osmotolerance.

Glucose/Sorbosone dehydrogenase (G*SDH*) is responsible for the production of NADPH through oxidative cleavage of glucose (Oubrie et al., 1999). Under salt stress condition, NADPH acts as reducing potential for output of reduced glutathione (GSH) and involved in activity of membrane bound NADPH oxidase, which results in accumulation of hydrogen peroxide (H_2_O_2_) (Wang et al., 2008). H_2_O_2_ acts as a signal in regulating G6PDH activity and expression of this enzyme in the glutathione cycle through which the ability of GSH regeneration was increased under salt stress (Wang et al., 2008). Thus, G6PDH plays a critical role in maintaining cellular GSH levels under long-term salt stress conditions (Wang et al., 2008). It indicates that *GSDH* of *pSSR4* is involved in salt stress tolerance possibly through the cytosolic accumulation of reducing equivalents (NADPH and GSH).

While in case of *pSSR6* only one *ORF* was identified, sharing homology with Serine/Threonine kinases. This encoded protein also possesses two conserved domains for the protein family STKc_PknB, i.e. the catalytic domain of bacterial Serine/Threonine kinases, PknB and similar proteins; STKs and TOMM_kin_cyc, i.e., TOMM system kinase/cyclase fusion protein. STKs are well known for activating genetic locus concerned with osmosensing (Hatzios et al., 2013) and inducing topological changes such as DNA supercoiling (Gupta et al., 2014). These osmosensing signal and DNA supercoiling could initiate uptake of the osmolyte glycine betaine, proline (Csonka, 1989) or initiates the expression of genetic elements required to cope with osmotic stress (Higgins et al., 1988). This could be the possible mechanism by which putative STK_PknB of *pSSR6* might be extending osmotolerance to the host *E. coli*.

The *pSSR21 ORF2* encodes a protein sharing homology with a hypothetical protein of *Candidatus Handelsmanbacteria* possessing a conserved domain for DUF3445 superfamily, i.e., an uncharacterized protein family having conserved RLP sequence motif (Bateman et al., 2010). However, its physiological characterization and intracellular ion concentration analysis indicates that it extends host osmotolerance property by maintaining low intracellular ion concentration even in the presence of an ionic stressor. However, a detailed mechanism still needs to be elucidated.

## Conclusion

In this study, the functional metagenomic approach was used to decipher salt stress tolerant genes in the saline soil microbiome. Identification of salt tolerant genes *BCAA_ABCtp, GSDH STK_Pknb*, and *duf3445* has enriched our understanding about the survivability and adaptability of microbes in the highly saline soil ecosystem. These salt tolerant genes can be used for crop improvement and for producing bioactive molecules under high salt conditions, which reduces the chances of contamination by other microbes.

## Author contributions

NC, MV: Designed the project; MV, VA: Performed experiments and NGS sequencing; SG, VM, and VA: Performed data analyses; MV, VM, and NC: Wrote the manuscript. All authors have read and approve the manuscript.

### Conflict of interest statement

The authors declare that the research was conducted in the absence of any commercial or financial relationships that could be construed as a potential conflict of interest.
