# Supplementary material for: Metagenomic Profiling of Soil Microbes to Mine Salt Stress Tolerance Genes
Source: Front Microbiol. 2018 Feb 8;9:159. doi: 10.3389/fmicb.2018.00159 (PMC5809485; doi:10.3389/fmicb.2018.00159)
Supplement: Supplementary file 1 [file DataSheet1.PDF]

Metagenomic profiling of soil microbes to mine salt stress tolerance genes

Authors

Vasim Ahmed[1,¶], Manoj Kumar Verma[1,¶], Shashank Gupta[1], Vibha Mandhan[1], Nar Singh Chauhan[1*,]

[¶]These authors contributed equally

[1]*Department of Biochemistry, Maharshi Dayanand University, Rohtak, Haryana, India*

*Corresponding Author

Dr N S Chauhan,

Department of Biochemistry, Maharshi Dayanand University, Rohtak, Haryana, India

Email: nschauhan@mdurohtak.ac.in

(A) Supplementary methods

A. Metagenomic DNA extraction: A 5 gram of soil sample was suspended in 14.5 ml of lysis buffer containing 1% (w/v) cetyl trimethyl ammonium bromide (CTAB), 100 mM of ethylenediaminetetraacetic acid (EDTA), 1.5 M of NaCl, 100 mM of Na_3PO_4, and 100 mM of Tris-HCl (pH 8.0). The mixture was incubated at 37 °C for 1 h with gentle shaking after adding Proteinase K (1 mg/g of the pellet). SDS was added to a final concentration of 2% and tubes were incubated again at 65°C for 2 h with occasional shaking. Lysate was centrifuged at 8000g for 10 min at ambient room temperature after addition of equal volume chloroform and isoamyl alcohol (24:1). The DNA was precipitated by 0.6 V of isopropyl alcohol and kept undisturbed for 1 h. The DNA was pelleted by centrifugation at 10,000g for 20 min and washed with 5 ml of 70% ethanol. The pellet was air dried and dissolved in 1 ml of Tris-EDTA buffer, pH 8.0. DNA was further purified on 0.7% low melting agarose and high molecular weight DNA was collected from the gel for library construction (Chauhan et al., 2009).

B. Metagenomic library construction: Four microgram of high molecular weight soil metagenomic DNA was partially digested with *Sau*3A1. Partially digested DNA fragments of approximately 2-10 kb were gel eluted using QIAquick Gel Extraction Kit (Qiagen, USA). Two hundred nanogram of partially digested soil metagenomic DNA was ligated to *Bam*HI digested and dephosphorylated *pUC*19 vector. The ligated product was electroporated at 200 Ω, 25 µf, and 12.5 kV/cm in *E. coli* DH10B using Micropulser II (Bio-Rad, USA). Recombinant transformants were selected on LB agar medium supplemented with ampicillin (100µg/ml). Randomly selected twenty recombinant clones were checked for insert size to calculate average insert size of the metagenomic library.

C. SSU rRNA gene amplification: Bacterial 16S rRNA genes were amplified from soil metagenomic DNA using the forward primer (5-CCATCTCATCCCTGCGTGTCTCCGACT CAGACGAGTGCGTGAGTTTGATCCTGGCTCAG-3), which contained the 454 Life Sciences primer B sequence, bacterial primer 8-27F, a unique 10nt multiplex identifier (ACGAGTGCGT), and the reverse primer (5-CGTATCGCCTCCCTCGCGCCATCAGGGAC TACCAGG GTATCT AA-3′), which contained the 454 Life Sciences primer A sequence and the broad-range bacterial primer 788–806R. SSU rRNA gene amplification was performed in a 50µl reaction mixture SSU rRNA gene amplification was performed in a 50µl reaction mixture (1x PCR Buffer (Fermentas, USA), 0.4 µM primers , template DNA (1ng/µl), 200mM of each dNTP (Fermentas, USA), 2mM $MgCl_2$, 1 U Taq DNA polymerase (Fermentas, USA) using PCR system (PeqLab, Germany). Polymerase chain reaction (PCR) condition was optimized at amplification was performed with an initial denaturation for 5 min. at 94°C and 30 cycles of 94°C for 1 min., 58°C for 1.30 min. and 72°C for 2.30 min. followed by a final extension at 72°C for 10 min.

(B) Supplementary Figure

Supplementary Figure S1. Minimum inhibitory concentration (MIC) analysis of salt stress tolerant clones. MIC of *E.coli* (DH10B) metagenomic clones SSR1 (●), SSR4 (▲), SSR6 (▼), SSR21(◀) and *E.coli* (DH10B) host strain carrying empty plasmid vector (■) at different concentration of NaCl (**A**) and KCl (**B**).

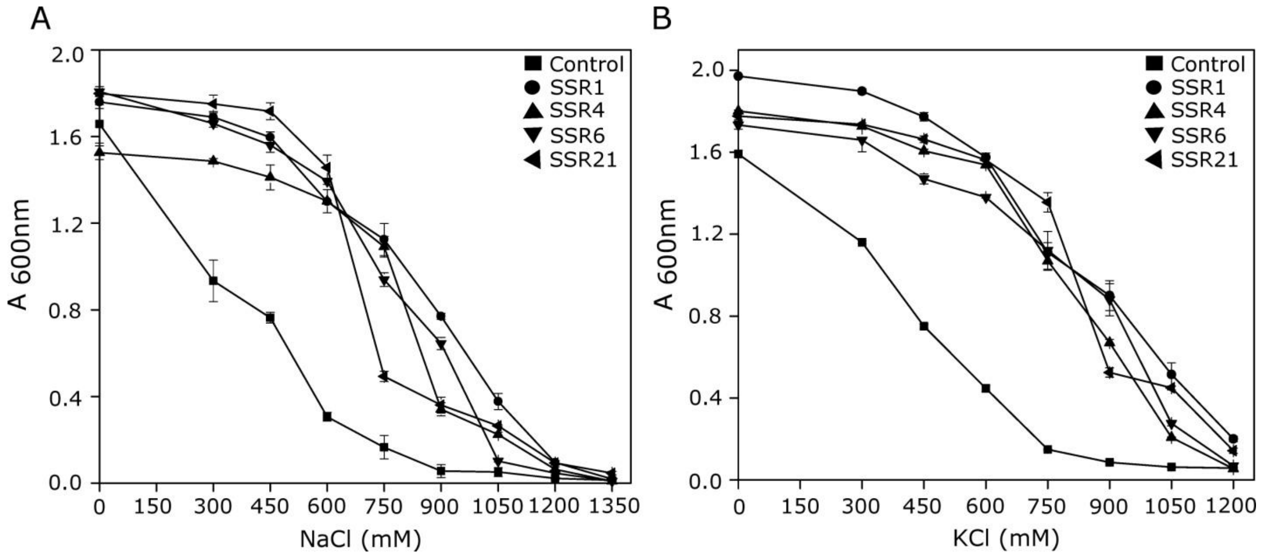

(C) **Supplementary Tables**

Supplementary Table S1. Oligonucleotide sequences used in present study

Sr.No	Primer ID	Primer sequence (5'-3')	Length (base)
**1.**	ORF 1 Fwd	GATCGCGCGCGCCCTGATG	19
**2.**	ORF 1 Rev	CTCGTAGAGCCACGGATAC	19
**3.**	ORF 4 Fwd	CTGAATTCATGACGCTGCTTGGAC	24
**4.**	ORF 4 Rev	CTAAGCTTTCAAACGTCCCGCCGG	24
**5.**	ORF 6 Fwd	GCGGAGGCTCATGTTGTG	18
**6.**	ORF 6 Rev	GTGCGCAAGGCATCGGTC	18
**7.**	ORF 21 Fwd	GAGATGGTAAGCGAGATG	18
**8.**	ORF 21 Rev	GAATCTATGCGGCGCTGTC	19

Supplementary Table S2: Various OTUs identified within saline soil microbome

OTUID	Abundance	Phylum	*Class*	*Order*	*Family*	*Genus*	*Species*
**780100**	**8**	Acidobacteria	*Solibacteres*	*Solibacterales*	*PAUC26f*		
**1117598**	**1**	Acidobacteria	*Solibacteres*	*Solibacterales*	*PAUC26f*		
**825569**	**2**	Acidobacteria	*Solibacteres*	*Solibacterales*	*Solibacteraceae*	*Candidatus Solibacter*	
**4476744**	**1**	Acidobacteria	*Solibacteres*	*Solibacterales*	*Solibacteraceae*	*Candidatus Solibacter*	
**564174**	**3**	Actinobacteria	*Acidimicrobiia*	*Acidimicrobiales*			
**970074**	**27**	Actinobacteria	*Acidimicrobiia*	*Acidimicrobiales*			
**4313541**	**2**	Actinobacteria	*Acidimicrobiia*	*Acidimicrobiales*			
**1115682**	**9**	Actinobacteria	*Acidimicrobiia*	*Acidimicrobiales*			
**827008**	**3**	Actinobacteria	*Acidimicrobiia*	*Acidimicrobiales*			
**508790**	**5**	Actinobacteria	*Acidimicrobiia*	*Acidimicrobiales*			
**835758**	**17**	Actinobacteria	*Acidimicrobiia*	*Acidimicrobiales*			
**1124227**	**4**	Actinobacteria	*Acidimicrobiia*	*Acidimicrobiales*			
**566820**	**3**	Actinobacteria	*Acidimicrobiia*	*Acidimicrobiales*			
**169682**	**4**	Actinobacteria	*Acidimicrobiia*	*Acidimicrobiales*	*koll13*		
**246078**	**1**	Actinobacteria	*Actinobacteria*	*Actinomycetales*	*Mycobacteriaceae*	*Mycobacterium*	
**236820**	**1**	Actinobacteria	*Actinobacteria*	*Actinomycetales*	*Pseudonocardiaceae*	*Amycolatopsis*	
**837069**	**1**	Actinobacteria	*Actinobacteria*	*Actinomycetales*	*Streptomycetaceae*	*Streptomyces*	
**608575**	**1**	Actinobacteria	*MB-A2-108*				
**4461164**	**2**	Actinobacteria	*MB-A2-108*	*0319-7L14*			
**4361436**	**2**	Actinobacteria	*MB-A2-108*	*0319-7L14*			
**1111090**	**14**	Actinobacteria	*Nitriliruptoria*	*Nitriliruptorales*	*Nitriliruptoraceae*		
**1127842**	**3**	Actinobacteria	*Nitriliruptoria*	*Nitriliruptorales*	*Nitriliruptoraceae*		
**4316723**	**1**	Actinobacteria	*Nitriliruptoria*	*Nitriliruptorales*	*Nitriliruptoraceae*		
**834349**	**2**	Actinobacteria	*Nitriliruptoria*	*Nitriliruptorales*	*Nitriliruptoraceae*		
**651077**	**1**	Actinobacteria	*Thermoleophilia*	*Gaiellales*	*Gaiellaceae*		

**4329468**	**1**	Bacteroidetes	*Cytophagia*	*Cytophagales*	*Flammeovirgaceae*	
**4385825**	**2**	Bacteroidetes	*Cytophagia*	*Cytophagales*	*Flammeovirgaceae*	
**278936**	**12**	Bacteroidetes	*Cytophagia*	*Cytophagales*	*Flammeovirgaceae*	*Fulvivirga*
**828978**	**2**	Bacteroidetes	*Cytophagia*	*Cytophagales*	*Flammeovirgaceae*	*Fulvivirga*
**1109032**	**5**	Bacteroidetes	*Flavobacteriia*	*Flavobacteriales*		
**834271**	**3**	Bacteroidetes	*Flavobacteriia*	*Flavobacteriales*	*Flavobacteriaceae*	
**4464306**	**14**	Bacteroidetes	*Flavobacteriia*	*Flavobacteriales*	*Flavobacteriaceae*	
**825539**	**1**	Bacteroidetes	*Flavobacteriia*	*Flavobacteriales*	*Flavobacteriaceae*	*Gramella*
**1104951**	**5**	Bacteroidetes	*Rhodothermi*	*Rhodothermales*	*Balneolaceae*	*KSA1*
**1107052**	**5**	Bacteroidetes	*Rhodothermi*	*Rhodothermales*	*Balneolaceae*	*KSA1*
**4414353**	**2**	Bacteroidetes	*Rhodothermi*	*Rhodothermales*	*Balneolaceae*	*KSA1*
**1106022**	**2**	Bacteroidetes	*Rhodothermi*	*Rhodothermales*	*Balneolaceae*	*KSA1*
**1100261**	**1**	Bacteroidetes	*Rhodothermi*	*Rhodothermales*	*Balneolaceae*	*KSA1*
**835013**	**1**	Bacteroidetes	*Rhodothermi*	*Rhodothermales*	*Balneolaceae*	*KSA1*
**832782**	**1**	Bacteroidetes	*Rhodothermi*	*Rhodothermales*	*Rhodothermaceae*	
**592121**	**8**	Bacteroidetes	*Rhodothermi*	*Rhodothermales*	*Rhodothermaceae*	
**4405435**	**9**	Bacteroidetes	*Rhodothermi*	*Rhodothermales*	*Rhodothermaceae*	
**831709**	**12**	Bacteroidetes	*Rhodothermi*	*Rhodothermales*	*Rhodothermaceae*	
**136597**	**1**	Bacteroidetes	*Saprospirae*	*Saprospirales*	*Chitinophagaceae*	
**4298545**	**1**	Bacteroidetes	*Saprospirae*	*Saprospirales*	*Chitinophagaceae*	
**1810798**	**1**	Firmicutes	*Bacilli*	*Bacillales*		
**295504**	**1**	Firmicutes	*Bacilli*	*Bacillales*	*Bacillaceae*	*Bacillus*
**4458917**	**1**	Firmicutes	*Bacilli*	*Bacillales*	*Bacillaceae*	*Bacillus*
**4474738**	**1**	Firmicutes	*Bacilli*	*Bacillales*	*Bacillaceae*	*Bacillus*
**3443340**	**2**	Firmicutes	*Bacilli*	*Bacillales*	*Bacillaceae*	*Bacillus*
**2579228**	**1**	Firmicutes	*Bacilli*	*Bacillales*	*Bacillaceae*	*Bacillus*
**686219**	**1**	Firmicutes	*Bacilli*	*Bacillales*	*Paenibacillaceae*	*Ammoniphilus*
**904452**	**1**	Firmicutes	*Bacilli*	*Bacillales*	*Staphylococcaceae*	
**366794**	**1**	Firmicutes	*Clostridia*	*Clostridiales*	*Ruminococcaceae*	
**350334**	**10**	Gemmatimonadetes				

**547148**	**1**	Gemmatimonadetes	*Gemm-1*			
**114055**	**1**	Gemmatimonadetes	*Gemm-1*			
**1141008**	**1**	Gemmatimonadetes	*Gemm-2*			
**2870419**	**4**	Gemmatimonadetes	*Gemm-2*			
**960862**	**2**	Gemmatimonadetes	*Gemm-2*			
**2000879**	**5**	Gemmatimonadetes	*Gemm-2*			
**4298777**	**21**	Gemmatimonadetes	*Gemm-2*			
**4323843**	**7**	Gemmatimonadetes	*Gemm-4*			
**1105601**	**2**	Gemmatimonadetes	*Gemmatimonadetes*			
**4476746**	**1**	Gemmatimonadetes	*Gemmatimonadetes*	*N1423WL*		
**242905**	**1**	Nitrospirae	*Nitrospira*	*Nitrospirales*	*Nitrospiraceae*	
**1106498**	**2**	Nitrospirae	*Nitrospira*	*Nitrospirales*	*Nitrospiraceae*	*Nitrospira*
**920745**	**4**	Proteobacteria	*Alphaproteobacteria*			
**264451**	**2**	Proteobacteria	*Alphaproteobacteria*			
**1002009**	**3**	Proteobacteria	*Alphaproteobacteria*			
**313798**	**2**	Proteobacteria	*Alphaproteobacteria*			
**558593**	**1**	Proteobacteria	*Alphaproteobacteria*			
**668527**	**14**	Proteobacteria	*Alphaproteobacteria*			
**304307**	**1**	Proteobacteria	*Alphaproteobacteria*	*Kiloniellales*		
**112782**	**1**	Proteobacteria	*Alphaproteobacteria*	*Kiloniellales*	*Kiloniellaceae*	*Thalassospira*
**273429**	**2**	Proteobacteria	*Alphaproteobacteria*	*Rhizobiales*		
**563408**	**1**	Proteobacteria	*Alphaproteobacteria*	*Rhizobiales*		

**1138879**	**1**	Proteobacteria	*Alphaproteobacteria*	*Rhizobiales*			
**593689**	**4**	Proteobacteria	*Alphaproteobacteria*	*Rhizobiales*	*Hyphomicrobiaceae*	*Devosia*	
**112992**	**1**	Proteobacteria	*Alphaproteobacteria*	*Rhizobiales*	*Hyphomicrobiaceae*	*Rhodoplanes*	
**150180**	**1**	Proteobacteria	*Alphaproteobacteria*	*Rhizobiales*	*Phyllobacteriaceae*		
**539878**	**1**	Proteobacteria	*Alphaproteobacteria*	*Rhizobiales*	*Phyllobacteriaceae*	*Mesorhizobium*	
**4321652**	**2**	Proteobacteria	*Alphaproteobacteria*	*Rhizobiales*	*Phyllobacteriaceae*	*Nitratireductor*	*pacificus*
**614651**	**1**	Proteobacteria	*Alphaproteobacteria*	*Rhizobiales*	*Rhizobiaceae*	*Agrobacterium*	
**4403263**	**1**	Proteobacteria	*Alphaproteobacteria*	*Rhodobacterales*	*Rhodobacteraceae*		
**4320404**	**1**	Proteobacteria	*Alphaproteobacteria*	*Rhodospirillales*	*Rhodospirillaceae*		
**775737**	**3**	Proteobacteria	*Alphaproteobacteria*	*Rhodospirillales*	*Rhodospirillaceae*		
**4382273**	**1**	Proteobacteria	*Alphaproteobacteria*	*Rhodospirillales*	*Rhodospirillaceae*		
**256400**	**12**	Proteobacteria	*Alphaproteobacteria*	*Rhodospirillales*	*Rhodospirillaceae*		
**544803**	**1**	Proteobacteria	*Alphaproteobacteria*	*Rhodospirillales*	*Rhodospirillaceae*		
**1115495**	**7**	Proteobacteria	*Alphaproteobacteria*	*Rhodospirillales*	*Rhodospirillaceae*		
**250348**	**4**	Proteobacteria	*Alphaproteobacteria*	*Rhodospirillales*	*Rhodospirillaceae*		
**210865**	**1**	Proteobacteria	*Alphaproteobacteria*	*Rhodospirillales*	*Rhodospirillaceae*		
**4305126**	**1**	Proteobacteria	*Alphaproteobacteria*	*Rickettsiales*			
**584900**	**1**	Proteobacteria	*Alphaproteobacteria*	*Sphingomonadales*	*Erythrobacteraceae*		
**153621**	**1**	Proteobacteria	*Alphaproteobacteria*	*Sphingomonadales*	*Erythrobacteraceae*		
**582344**	**1**	Proteobacteria	*Alphaproteobacteria*	*Sphingomonadales*	*Erythrobacteraceae*		
**1027418**	**1**	Proteobacteria	*Alphaproteobacteria*	*Sphingomonadales*	*Erythrobacteraceae*		
**161513**	**2**	Proteobacteria	*Alphaproteobacteria*	*Sphingomonadales*	*Erythrobacteraceae*	*Erythrobacter*	
**421125**	**1**	Proteobacteria	*Alphaproteobacteria*	*Sphingomonadales*	*Erythrobacteraceae*	*Lutibacterium*	
**831826**	**1**	Proteobacteria	*Alphaproteobacteria*	*Sphingomonadales*	*Sphingomonadaceae*		
**582973**	**1**	Proteobacteria	*Alphaproteobacteria*	*Sphingomonadales*	*Sphingomonadaceae*		
**209920**	**1**	Proteobacteria	*Betaproteobacteria*				
**4345237**	**1**	Proteobacteria	*Betaproteobacteria*				
**4443757**	**1**	Proteobacteria	*Betaproteobacteria*	*Burkholderiales*	*Oxalobacteraceae*		
**509402**	**6**	Proteobacteria	*Betaproteobacteria*	*MND1*			
**813885**	**1**	Proteobacteria	*Betaproteobacteria*	*MND1*			

**1113290**	**1**	Proteobacteria	*Betaproteobacteria*	*MND1*		
**242612**	**1**	Proteobacteria	*Betaproteobacteria*	*MND1*		
**577068**	**1**	Proteobacteria	*Betaproteobacteria*	*MND1*		
**218246**	**1**	Proteobacteria	*Betaproteobacteria*	*MND1*		
**38503**	**1**	Proteobacteria	*Betaproteobacteria*	*Nitrosomonadales*	*Nitrosomonadaceae*	
**4470827**	**8**	Proteobacteria	*Deltaproteobacteria*	*GMD14H09*		
**259881**	**1**	Proteobacteria	*Deltaproteobacteria*	*Myxococcales*		
**4300036**	**1**	Proteobacteria	*Deltaproteobacteria*	*Myxococcales*		
**812224**	**1**	Proteobacteria	*Deltaproteobacteria*	*Myxococcales*	*Haliangiaceae*	
**1146177**	**1**	Proteobacteria	*Deltaproteobacteria*	*Myxococcales*	*Haliangiaceae*	
**4474780**	**1**	Proteobacteria	*Deltaproteobacteria*	*Myxococcales*	*Haliangiaceae*	
**1115600**	**1**	Proteobacteria	*Deltaproteobacteria*	*Myxococcales*	*Nannocystaceae*	*Plesiocystis*
**4363694**	**1**	Proteobacteria	*Deltaproteobacteria*	*Spirobacillales*		
**552030**	**1**	Proteobacteria	*Deltaproteobacteria*	*Syntrophobacterales*	*Syntrophobacteraceae*	
**1121555**	**1**	Proteobacteria	*Deltaproteobacteria*	*Syntrophobacterales*	*Syntrophobacteraceae*	
**981968**	**1**	Proteobacteria	*Deltaproteobacteria*	*Syntrophobacterales*	*Syntrophobacteraceae*	
**4456337**	**4**	Proteobacteria	*Deltaproteobacteria*	*Syntrophobacterales*	*Syntrophobacteraceae*	
**1107752**	**1**	Proteobacteria	*Deltaproteobacteria*	*Syntrophobacterales*	*Syntrophobacteraceae*	
**1106493**	**10**	Proteobacteria	*Gammaproteobacteria*			
**825722**	**2**	Proteobacteria	*Gammaproteobacteria*			
**4456120**	**2**	Proteobacteria	*Gammaproteobacteria*			
**354247**	**1**	Proteobacteria	*Gammaproteobacteria*	*Alteromonadales*	*Alteromonadaceae*	
**836140**	**1**	Proteobacteria		*Alteromonadales*	*Alteromonadaceae*	

			Gammaproteobacteria			
**137110**	**1**	Proteobacteria		*Alteromonadales*	*Alteromonadaceae*	*Microbulbifer*
			Gammaproteobacteria			
**975849**	**38**	Proteobacteria		*Chromatiales*		
			Gammaproteobacteria			
**1117949**	**4**	Proteobacteria		*Chromatiales*		
			Gammaproteobacteria		*Ectothiorhodospiraceae*	
**4402142**	**3**	Proteobacteria		*Chromatiales*		
			Gammaproteobacteria		*Ectothiorhodospiraceae*	
**1852581**	**2**	Proteobacteria		*Chromatiales*		
			Gammaproteobacteria		*Ectothiorhodospiraceae*	
**1113940**	**1**	Proteobacteria		*Chromatiales*		
			Gammaproteobacteria		*Ectothiorhodospiraceae*	
**1111983**	**2**	Proteobacteria		*Marinicellales*	*Marinicellaceae*	
			Gammaproteobacteria			
**4329768**	**1**	Proteobacteria		*Oceanospirillales*		
			Gammaproteobacteria			
**972234**	**1**	Proteobacteria		*Oceanospirillales*	*Halomonadaceae*	
			Gammaproteobacteria			
**1106355**	**1**	Proteobacteria		*Oceanospirillales*	*Halomonadaceae*	
			Gammaproteobacteria			
**3270407**	**1**	Proteobacteria		*Oceanospirillales*	*Oceanospirillaceae*	
			Gammaproteobacteria			
**527669**	**3**	Proteobacteria		*Salinisphaerales*	*Salinisphaeraceae*	
			Gammaproteobacteria			
**244546**	**2**	Proteobacteria		*Salinisphaerales*	*Salinisphaeraceae*	
			Gammaproteobacteria			
**589587**	**2**	Proteobacteria		*Thiotrichales*	*Piscirickettsiaceae*	
			Gammaproteobacteria			
**4432131**	**1**	Proteobacteria		*Thiotrichales*	*Piscirickettsiaceae*	
			Gammaproteobacteria			
**1070610**	**3**	Proteobacteria		*Thiotrichales*	*Piscirickettsiaceae*	
			Gammaproteobacteria			

**904578**	**1**	Proteobacteria	*Gammaproteobacteria*	*Thiotrichales*	*Piscirickettsiaceae*	
**278985**	**1**	Proteobacteria	*Gammaproteobacteria*	*Thiotrichales*	*Piscirickettsiaceae*	*Methylophaga*
**527397**	**1**	Proteobacteria	*Gammaproteobacteria*	*Xanthomonadales*	*Sinobacteraceae*	
**320782**	**2**	Proteobacteria	*Gammaproteobacteria*	*Xanthomonadales*	*Sinobacteraceae*	*Steroidobacter*
**731073**	**1**	Proteobacteria	*Gammaproteobacteria*	*Xanthomonadales*	*Sinobacteraceae*	*Steroidobacter*
**818495**	**1**	Proteobacteria	*Gammaproteobacteria*	*Xanthomonadales*	*Xanthomonadaceae*	*Luteimonas*
**1143681**	**3**	Proteobacteria	*Gammaproteobacteria*	*Xanthomonadales*	*Xanthomonadaceae*	*Lysobacter*
